# Beyond the snapshot: continuous optical surface monitoring reveals non-linear temporal dynamics and postural stability window in free-breathing breast radiotherapy

**DOI:** 10.3389/fonc.2026.1916923

**Published:** 2026-09-03

**Authors:** Wenlingjie Huang, Ying Peng, Tiandi Zhao, Jinna Li, Haitao Sun, Ruilin Zhang, Jiashuang Zhang, Ping Jiang, Junjie Wang

**Affiliations:** 1 Department of Radiation Oncology, Peking University Third Hospital, Beijing, China; 2 Department of General Surgery, Peking University Third Hospital, Beijing, China

**Keywords:** AlignRT, breast cancer radiotherapy, intrafractional motion, non-linear temporal dynamics, postural stability window

## Abstract

**Purpose:**

This study aimed to evaluate the clinical consistency between AlignRT surface monitoring and fan-beam computed tomography (FBCT)–based single-timepoint volumetric verification in free breathing (FB) breast radiotherapy, and to quantitatively characterize the nonlinear temporal dynamics of intrafractional motion.

**Materials and methods:**

A retrospective analysis was conducted on 485 FB volumetric modulated arc therapy (VMAT) fractions from 38 patients treated following breast conserving surgery. Intrafractional motion was monitored concurrently using FBCT (endpoint positional snapshots) and AlignRT (continuous six degree of freedom tracking). Raw surface signals were decomposed into baseline drift and respiratory motion components using a 0.05 Hz zero phase low pass filter. Linear mixed effects models (LMMs) and segmented regression analyses were employed to evaluate clinical predictors and characterize temporal stability patterns.

**Results:**

Both AlignRT and FBCT demonstrated excellent agreement along the lateral axis (median difference, 0.021 mm). In contrast, the vertical axis exhibited opposing directional shifts, in which FBCT indicated a slight anterior displacement (0.06 mm), whereas AlignRT detected a posterior drift (−0.17 mm), likely reflecting differences between internal bony expansion and external soft tissue deformation. LMM analysis revealed that higher respiratory frequency was significantly associated with reduced motion amplitudes. Segmented regression identified a multiphasic temporal trajectory, including an observed “stability window” between 100.7 s and 138.8 s, after which postural degradation significantly increased.

**Conclusions:**

AlignRT provides an indispensable continuous-tracking supplement to single-timepoint FBCT. A shallow, rapid breathing pattern and treatment delivery within the ~100-140s stability window may help optimize posture maintenance. Continuous surface monitoring may therefore play a critical role in maintaining dosimetric accuracy during extended treatment delivery.

## 1 Introduction

Post-breast-conserving surgery radiotherapy is one of the standard modalities in the comprehensive treatment of early-stage breast cancer. An authoritative meta-analysis of tens of thousands of patients conducted by the Early Breast Cancer Trialists’ Collaborative Group (EBCTCG) demonstrated the benefit of adjuvant radiotherapy after breast-conserving surgery (BCS) (1). It approximately halved the 10-year recurrence risk and significantly reduced 15-year breast cancer–specific mortality. These findings firmly established radiotherapy as a fundamental component of breast-conserving therapy (1). In recent years, the widespread application of advanced technologies, such as volumetric modulated arc therapy (VMAT), has remarkably enhanced target dose conformity and improved the sparing of organs at risk (OARs), including the heart and ipsilateral lung (2). However, these technological advancements also impose notably more stringent requirements on patient setup precision and intra-fractional motion management, as geometric inaccuracies can directly result in inadequate dose coverage of the planning target volume (PTV), thereby compromising the efficacy of the originally defined margin strategy (3, 4). Under free-breathing (FB) conditions, patients’ intra-fractional motion not only includes periodic respiratory fluctuations, but also involves postural baseline drift that gradually accumulates over time (5, 6). Without continuous and effective monitoring and intervention, this dynamic displacement will severely undermine the dosimetric advantages of high-precision radiotherapy (7).

At present, image-guided technologies, such as fan-beam computed tomography (FBCT) or cone-beam CT (CBCT), remain the clinical gold standard for verifying internal bony anatomy (8). However, traditional imaging verification has inherent limitations of delivering additional ionizing radiation to patients, and they can provide a “single-timepoint snapshot” before or at the end of treatment, failing to reflect dynamic errors during treatment (9, 10). In contrast, surface-guided radiation therapy (SGRT), such as AlignRT, has driven a transformative advancement in radiotherapy positioning. Multiple studies have confirmed that it cannot only improve the traditional “three-point tattoo” method and significantly reduce setup errors, as further confirmed by recent multicenter and randomized crossover evidence in breast cancer radiotherapy (11–13), but also enables radiation-free four-dimensional monitoring of external surface motion through high-frame-rate six-degree-of-freedom (6DoF) tracking (14–16). The target volume in breast radiotherapy (e.g., the whole breast or chest wall) lies close to the skin surface. Optical surface imaging may therefore reflect the true motion and deformation of the target more directly than conventional IGRT, which relies only on internal bony landmarks (10, 15). Although the application of SGRT in clinics is increasingly popular, under complex FB conditions, the consistency between its continuous tracking capability and traditional FBCT single-timepoint volumetric verification, as well as the precise extent to which continuous surface monitoring provides incremental and complementary value to conventional radiologic verification, still require confirmation through high-quality clinical evidence (3, 17–19).

Furthermore, most existing studies on intrafractional motion often use simple averaging methods to quantify overall errors, frequently ignoring the complex temporal dynamics of the motion itself (20). Physiological and clinical outcomes indicated that when patients are required to maintain an unnatural arms-up position, their superficial soft tissues become highly susceptible to gravitational sagging and postural degradation secondary to muscle fatigue (4, 20, 21). Moreover, previous studies have demonstrated that baseline drift may begin to develop during the early phase of treatment, reflecting initial postural adaptation and relaxation processes (4, 21). Recent studies have also pointed out that the delivery time of treatment techniques (e.g., rapidly modulated VMAT versus prolonged static IMRT) has a decisive impact on the accumulation of intra-fractional displacement (6, 22). Despite the increasing clinical research of SGRT, several fundamental questions regarding intra-fraction motion remain unresolved. In particular, the temporal evolution of intra-fractional drift and the existence of a physiological “stability window” during free-breathing radiotherapy remain poorly understood. Furthermore, the biomechanical influence of different respiratory patterns, such as shallow or deep breathing, on patient displacement has not been quantitatively characterized. Robust quantitative evidence addressing these phenomena remains limited, restricting the development of evidence-based strategies for real-time motion monitoring and intervention.

Based on this background, this study aimed to systematically evaluate the clinical consistency between AlignRT continuous monitoring and FBCT single-timepoint volumetric verification in patients undergoing FB radiotherapy post-BCS, and to clarify the indispensable complementary value of continuous surface tracking for single radiological verification. Meanwhile, this study innovatively presented high-fidelity signal decoupling technology, aiming to accurately reveal the non-linear temporal dynamics of intra-fractional motion, and to quantify the “stability window” of posture maintenance, as well as the constraining effect of breathing patterns on spatial displacement. Furthermore, because SGRT and radiographic imaging interrogate fundamentally different anatomical references—the external soft-tissue surface versus the internal bony skeleton—we hypothesized that these two structures may not move in a strictly coupled manner during free-breathing treatment. Specifically, we postulated that gravitational soft-tissue relaxation and postural changes could drive the external surface in a direction that diverges from, or is even opposite to, that of the underlying bony anatomy, most notably along the vertical axis. Explicitly testing this “anatomical decoupling” hypothesis, and characterizing its magnitude and direction, therefore constitutes a central aim of the present study. The findings of this study will provide a theoretical basis for developing more scientific and precise real-time monitoring and intervention strategies, while advancing the mechanistic understanding of internal–external motion coupling in surface-guided radiotherapy.

## 2 Materials and methods

### 2.1 Patients’ selection and treatment protocol

This retrospective study was conducted at the Department of Radiation Oncology, Peking University Third Hospital. This study was approved by the Institutional Review Board of our institution (Approval No.: IRB00006761-M20260217), and it was conducted in accordance with the Declaration of Helsinki. Due to the retrospective nature of the study, the requirement for written informed consent was waived. Data were collected from May 7, 2025, to November 4, 2025, involving an initial cohort of 41 female patients who had undergone BCS and subsequently received whole-breast irradiation (WBI), with or without supraclavicular and subclavian nodal irradiation, using VMAT under FB conditions. A total of 687 treatment fractions were initially screened for inclusion in the study. Fractions were excluded according to stringent data quality criteria applied to both FBCT and AlignRT records. Specific reasons for exclusion included missing AlignRT data (104 fractions), incomplete scan logs (89 fractions), and mismatched timestamps between the two systems (9 fractions). The missing AlignRT recordings were primarily attributed to technical data management issues during the early implementation stage, in which raw tracking files were not properly saved during certain treatment sessions. 3 patients were entirely excluded because all their fractions failed the data-quality criteria. As a result, the final cohort comprised 38 patients (17 left-sided and 21 right-sided), involving 485 validated treatment fractions eligible for analysis (**Supplementary Figure 2**). Given the consistency in surgical procedures, planning techniques, and breathing protocols, data from left- and right-sided breast cancer cases were pooled for statistical analysis.

### 2.2 Motion monitoring and data acquisition

Intrafractional motion was monitored simultaneously using two distinct modalities. FBCT (United Imaging, Shanghai, China) scans were acquired both immediately after patient setup, promoting alignment and adjustment, and at the end of each treatment fraction to assess intrafractional positional deviations across the three translational dimensions: X (lateral), Y (longitudinal), and Z (vertical). These scans served as the comparative standard for positional verification. The acquisition duration was approximately 11–12 seconds, with slight variations depending on the scan length required for each patient’s anatomical coverage. Because this duration covered more than one respiratory cycle for most patients, the reconstructed images represented a time-averaged free-breathing anatomical state rather than a respiratory phase-specific reconstruction. Concurrently, the AlignRT system (VisionRT, London, UK) was employed for real-time optical surface monitoring, tracking the patient’s position in six degrees of freedom (6DoF): three translational (lateral [LAT], longitudinal [LNG], vertical [VRT]) and three rotational (yaw, roll, pitch) directions. To evaluate the tracking performance, three specific metrics were derived from the AlignRT data: the mean of real-time displacements, the mean baseline drift (calculated via motion decomposition), and the endpoint drift value recorded at the end of the fraction.

### 2.3 Assessment of setup accuracy and motion characteristics

To overall quantify intrafractional movement, the proportion of fractions exceeding predefined clinical thresholds (1 mm/1°, 2 mm/2°, and 3 mm/3°) was calculated for all translational and rotational axes. Systematic directional preferences in intrafractional motion were assessed using one-sample Wilcoxon signed-rank tests to evaluate deviations from zero. The magnitude of instability was determined by the root mean square (RMS) of these deviations. Differences in motion magnitude across the three translational and three rotational axes were analyzed using the non-parametric Friedman test, followed by *post-hoc* pairwise comparisons with Bonferroni correction to adjust for multiple testing.

### 2.4 Respiratory motion and baseline drift decomposition

To quantify the intrafractional motion characteristics, the raw surface-tracking signals acrosssix degrees of freedom (6DoF), denoted as *S(t)*, were decomposed into two distinct components: baseline drift *D(t)* and respiratory motion *R(t)*. This separation was achieved by employing a second-order zero-phase Butterworth low-pass filter with a cutoff frequency of 0.05 Hz (f_c_), corresponding to a 20-second period. This frequency threshold was selected to effectively isolate slow postural shifts from the more rapid, cyclic respiratory oscillations. Resting adult respiration typically occupies a band of approximately 0.20–0.33 Hz (12–20 breaths/min), and in the present cohort the measured respiratory frequency ranged from 0.143 to 0.437 Hz (median 0.292 Hz; see **Supplementary Figure 1** and baseline characteristics). Because even the slowest observed respiratory oscillation (0.143 Hz) remained approximately 2.9-fold above the 0.05 Hz cutoff, the entire respiratory band lay within the filter stop-band, whereas clinically relevant baseline drifts (frequencies well below 0.05 Hz) were preserved in the pass-band. This ensured a clean spectral separation between postural baseline drift D(t) and respiratory motion R(t). The baseline drift *D(t)* was defined as the filtered low-frequency signal (Equation 1):

(1) $$D\left(t\right)=\;LPF\;\left(S\left(t\right),\;f_{c}\;=\;0.05\;\text{Hz}\right)$$

**Figure 1:**
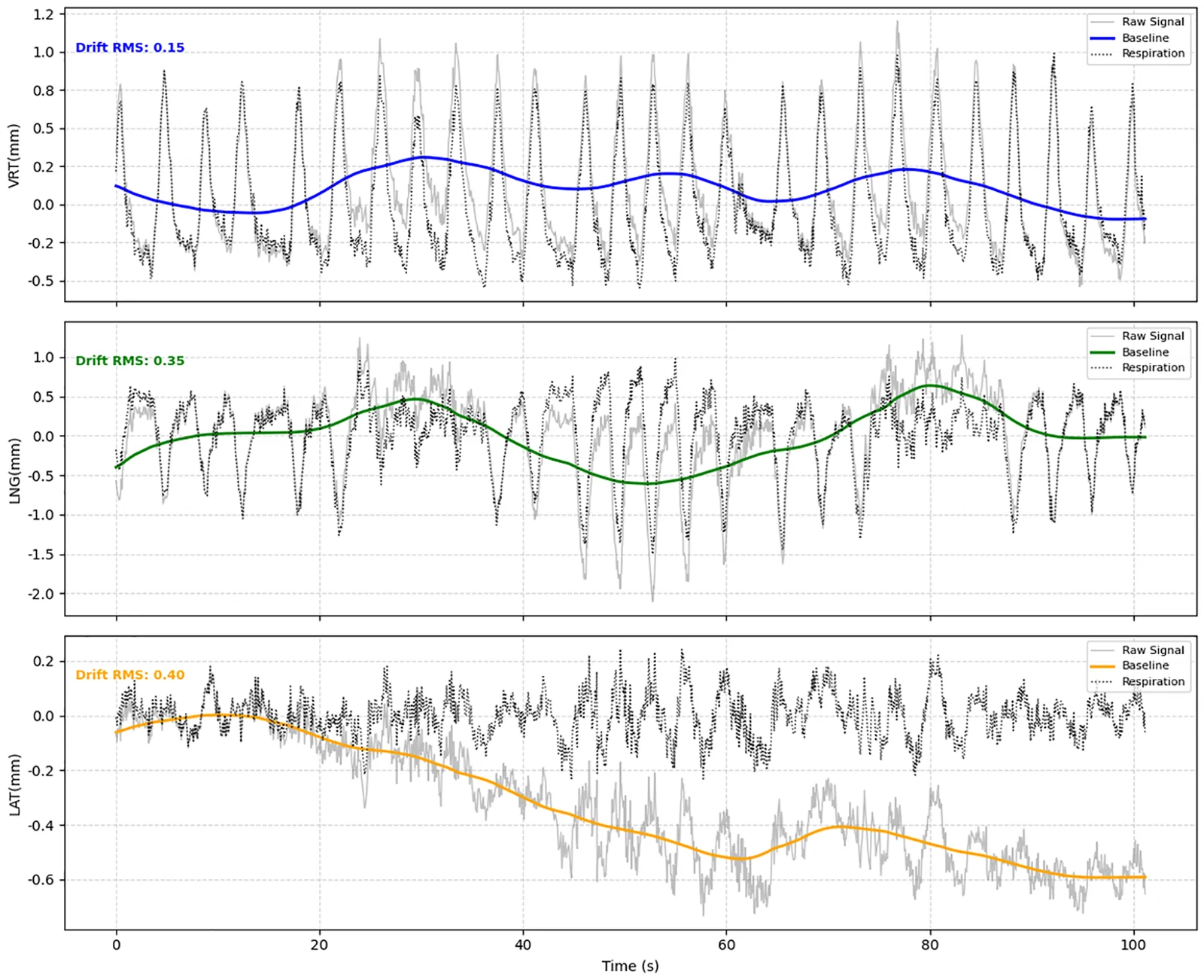
Representative example of intrafractional motion signal decomposition in the three translational degrees of freedom (3DoF). While the analysis was performed across all 6DoF, the translational axes (VRT, LNG, LAT) are shown here to illustrate the separation.

A typical example of intra-fractional motion signal decomposition is illustrated in **Figure 1**. The pure respiratory component *R(t)* was then derived by subtracting the baseline drift from the original raw motion data, as shown in Equation 2:

(2) R(t)= S(t)− D(t)

Respiratory motion magnitude was evaluated using two complementary metrics to capture different physical properties of the signal. The mean peak-to-peak amplitude was determined via a peak-finding algorithm that identified local extrema in the respiratory component, providing a direct measurement of the average physical excursion. Conversely, the respiratory root-mean-square (respiratory RMS) was calculated as the quadratic mean of the entire respiratory time-series to characterize the global energy and variance of the respiratory waveform. For a discrete time-series with N sampling points, the respiratory RMS was defined as shown in Equation 3:

(3) $$RMS_{res}=\sqrt{\frac{1}{N}\cdot \sum_{i=1}^{N}R{\left(t_{i}\right)}^{2}}$$

This distinction allows for a comprehensive assessment of both maximum spatial displacement and overall signal intensity.

The baseline drift was characterized using multiple parameters to evaluate setup stability and facilitate comparison with radiographic measurements. Beyond the standard drift RMS, quantifying cumulative deviation throughout the monitoring session, three specific metrics were identified for clinical validation: the real-time mean (average position of the raw signal), the mean drift (average position of the extracted baseline), and the endpoint drift (the final positional offset at the conclusion of monitoring).

### 2.5 Correlation and agreement between systems

To validate the optical surface monitoring against radiographical measurements, Spearman’s rank correlation coefficients were calculated between the FBCT endpoint measurements and the three AlignRT metrics (real-time mean, mean drift, and endpoint drift) across the lateral, longitudinal, and vertical axes. Differences between the monitoring metrics were evaluated using the Friedman test, while systematic deviations were quantified using Wilcoxon signed-rank test and Hodges-Lehmann median difference estimators. Clinical agreement between the two systems was further assessed using Bland-Altman analysis, which calculated the mean bias and 95% limits of agreement (LoA).

### 2.6 Multivariate analysis of factors influencing motion

To identify clinical predictors of intrafractional stability, multivariate linear mixed-effects models were developed, with patient identity included as a random intercept to account for inter-patient variability. Repeated fractions within each patient were modeled using an autoregressive correlation structure (AR1) to account for within-patient temporal correlation. These models assessed the impact of fixed effects, including treatment duration, respiratory frequency, patient age, laterality, and fraction number, on two dependent variables: the baseline drift RMS and the respiratory motion RMS. To ensure the robustness of the statistical inferences, an outlier detection and removal procedure was implemented prior to the final modeling of baseline and respiratory dynamics. Intrafractional motion metrics were screened for transient motion artifacts (e.g., coughing, swallowing, deep breaths, or sudden postural adjustments) that do not represent steady-state baseline drift and breathing patterns. Observations exceeding the threshold of mean ± 3 standard deviations (SD) were classified as extreme outliers and excluded from the LMMs and locally estimated scatterplot smoothing (LOESS) trend visualizations. This robust approach could mitigate the influence of a small number of high-magnitude, non-respiratory events that could otherwise disproportionately bias the regression slopes, thereby ensuring that the reported results more accurately represent the typical respiratory motion patterns of the patient cohort.

### 2.7 Temporal evolution and segmented regression analysis

The temporal evolution of intra-fractional motion was first visualized using locally estimated scatterplot smoothing (LOESS) to identify potential non-linear trajectories in both baseline drift and respiratory stability. To formally quantify these trends and detect the precise timing of stability degradation, segmented (piecewise) linear regression analysis was carried out using a non-linear least-squares algorithm. In contrast to traditional sensitivity tests with fixed candidate knots, the inflection point (T_break_) was treated as an unknown parameter that was estimated by the model. The regression was formulated by the Equation 4:

(4) $$Y\;=\;b_{0}\;+\;b_{1}\cdot \;t\;+\;b_{2}\cdot \;\left(t\;-\;T_{break}\right)\cdot \;I\left(t\;>\;T_{break}\right)$$

where *b_1_* represents the initial drift or respiration velocity, *b_2_* denotes the change in slope after the inflection point, and *I* is an indicator function. Based on the preliminary LOESS observations and clinical relevance, the search range for T_break_ was constrained between 100 s and 150 s according to the hints reflected by the LOESS figures. Statistical significance of the breakpoint was determined by evaluating 95% confidence intervals (CIs) of the slope change (*b_2_*); an interval that did not cross zero was interpreted as indicating a statistically significant acceleration or deceleration in motion magnitude.

The statistical analyses and non-linear modeling were performed using SPSS 26.0.0.1 software (IBM Corp., Armonk, NY, USA). All statistical tests were two-sided, and P<0.05 was considered statistically significant. Data visualization and graphical representations were generated using Python (version 3.12.7, packaged by Anaconda, Inc., Austin, TX, USA) with the Matplotlib (version 3.9.2) and Seaborn (version 0.13.2) libraries.

## 3 Results

### 3.1 Baseline characteristics

After excluding fractions with sub-optimal data quality, a total of 38 female patients withbreast cancer after receiving BCS were enrolled, covering 485 treatment fractions. The cohort included 17 patients with left-sided breast cancer (264 fractions) and 21 patients with right-sided breast cancer (221 fractions). The mean age of the cohort was 50.3 ± 10.4 years. All patients were treated using VMAT under FB conditions, in which simultaneous intra-fraction motion monitoring was performed by both AlignRT and FBCT. The average number of fractions per patient was 12.8 ± 5.8, and the mean treatment (monitoring) duration per fraction was 121 ± 30 s. The measured respiratory frequency across the cohort was 17.6 ± 3.1 breaths/min (median 17.5, IQR 3.6, range 8.6–26.2 breaths/min; equivalently 0.293 ± 0.052 Hz), the full distribution of which is presented in **Supplementary Figure 1**. Given the consistency in surgical procedures, planning techniques, and breathing protocols across the cohort, data from left- and right-sided cases were pooled for subsequent statistical analysis.

In terms of the proportion of setup deviations exceeding predefined thresholds, as presented in **Table 1**, the highest exceedance rates at the 1 mm/1° level were found in the translational directions, with VRT and LNG exhibiting comparable frequencies (14.43% and 14.05%, respectively), while LAT demonstrated a lower rate (4.32%). Exceedance rates significantly decreased at more stringent thresholds. For VRT, 2.68% and 1.40% of observations exceeded 2 mm/2° and 3 mm/3°, respectively; for LNG, the corresponding rates were 3.04% and 1.58%; and for LAT, 1.59% and 1.24%. Rotational deviations were generally infrequent, with exceedance rates at the 1° threshold of 1.52% for YAW, 0.77% for ROLL, and 1.96% for PITCH, decreasing to below 0.5% at the 2° threshold and below 0.3% at the 3° threshold. Overall, larger deviations were predominantly identified in translational direction rather than in rotational direction.

**Table 1:** Proportion of fractions exceeding predefined intra-fractional motion thresholds across six degrees of freedom (N = 485 fractions).

Direction	≥ 1 mm / 1°	≥ 2 mm / 2°	≥ 3 mm / 3°
Translational			
Vertical	14.43%	2.68%	1.40%
Longitudinal	14.05%	3.04%	1.58%
Lateral	4.32%	1.59%	1.24%
Rotational			
Yaw	1.52%	0.33%	0.28%
Roll	0.77%	0.24%	0.06%
Pitch	1.96%	0.48%	0.27%

### 3.2 Directional analysis

One-sample Wilcoxon signed-rank test was employed to determine whether the intrafraction deviations exhibited systematic directional preferences (**Table 2**). No significant directional bias was identified in the lateral (X) axis for either system (P = 0.413 and 0.369, respectively). In the longitudinal (Y) axis, AlignRT monitoring captured a statistically significant inferior drift (median H-L estimate = -0.07 mm, P< 0.001), whereas FBCT measurements exhibited no such trend (P = 0.211). In the vertical (Z) axis, both modalities demonstrated highly significant systematic shifts (P< 0.001), while in opposite directions. FBCT measurements indicated an anterior displacement (median = 0.06 mm), whereas AlignRT monitoring showed a posterior drift (median = −0.17 mm). Notably, despite their statistical significance, 95% CIs for these translational metrics were relatively wide, indicating remarkable inter-patient variability. AlignRT monitoring identified a significantly systematic negative Yaw rotation (median = -0.02°, P = 0.005). In contrast, no significant directional preferences were found for roll (P = 0.360) or pitch (P = 0.901) rotations, which remained predominantly stochastic at the population level.

**Table 2:** Directional analysis of intrafraction motion and AlignRT monitoring (N = 485).

Dimension	Metric	Median (H-L Est.)	95% CI	P-value	Conclusion
LAT (X)	FBCT	-0.00 mm	[-0.23, 0.18]	0.413	Non-significant
	AlignRT	0.01 mm	[-2.85, 2.64]	0.369	Non-significant
LNG (Y)	FBCT	0.01 mm	[-0.30, 0.26]	0.211	Non-significant
	AlignRT	**-0.07** mm	**[-0.97, 1.05]**	**< 0.001**	**Significantly Inferior**
VRT (Z)	FBCT	**0.06** mm	**[-0.14, 0.40]**	**< 0.001**	**Significantly Anterior**
	AlignRT	**-0.17** mm	**[-1.03, 1.93]**	**< 0.001**	**Significantly Posterior**
YAW	AlignRT	**-0.02°**	**[-0.37, 0.22]**	**0.005**	**Significantly Negative**
ROLL	AlignRT	0.00°	[-0.25, 0.20]	0.360	Non-significant
PITCH	AlignRT	0.00°	[-0.48, 0.25]	0.901	Non-significant

Bold values indicate statistically significant results (P < 0.05).

### 3.3 AlignRT & FBCT comparisons

Spearman’s rank correlation analysis was performed on 485 treatment fractions to evaluate the relationship between FBCT-measured endpoint displacements and the three monitoring metrics from AlignRT (real-time mean, mean baseline drift, and endpoint drift). As presented in **Table 3**, statistically significant correlations were found across all three dimensions (all P< 0.001). In the lateral (LAT) and longitudinal (LNG) axes, FBCT measurements exhibited weakly positive correlations with AlignRT metrics (r_s_ range, 0.167 to 0.278). In contrast, the vertical (VRT) axis exhibited a weakly negative correlation (r_s_​ range, -0.220 to -0.228). Notably, internal consistency in the AlignRT system was approximately perfect, with real-time means and mean baseline drifts exhibiting a correlation of r_s_​ > 0.99 (P< 0.001) across all the dimensions.

**Table 3:** Spearman’s rank correlation (r_s_​) between AlignRT and FBCT.

Dimension	Comparison pair	r_s_​	P
Lateral (X)	FBCT_X vs. Real-time Mean	0.226	< 0.001
	FBCT_X vs. Drift Mean	0.227	< 0.001
	FBCT_X vs. Endpoint Drift	0.227	< 0.001
Longitudinal (Y)	FBCT_Y vs. Real-time Mean	0.167	< 0.001
	FBCT_Y vs. Drift Mean	0.170	< 0.001
	FBCT_Y vs. Endpoint Drift	0.278	< 0.001
Vertical (Z)	FBCT_Z vs. Real-time Mean	-0.224	< 0.001
	FBCT_Z vs. Drift Mean	-0.228	< 0.001
	FBCT_Z vs. Endpoint Drift	-0.220	< 0.001

The systematic differences between FBCT endpoint measurements and AlignRT monitoring metrics were quantified using the Wilcoxon signed-rank test and the Hodges-Lehmann median difference estimator (**Table 4**). In the X axis, FBCT exhibited excellent agreement with AlignRT’s real-time mean and mean baseline drift, with a negligible median difference of 0.021 mm for both pairs (adjusted P = 1.000). However, a significant difference was identified between FBCT and the endpoint drift (median difference: 0.097 mm; 95% CI: [0.050, 0.148]; P = 0.001). In the Y axis, AlignRT metrics systematically deviated from FBCT measurements (all adjusted P< 0.001). The median differences for real-time mean, mean drift, and endpoint drift were -0.073 mm, -0.071 mm, and -0.130 mm, respectively. In the Z axis, this dimension demonstrated the most remarkable systematic offsets. AlignRT measurements were consistently larger than FBCT values, with median differences ranging from -0.235 mm to -0.351 mm (all adjusted P< 0.001).

**Table 4:** Pairwise comparisons of positional deviations between FBCT and AlignRT (median: AlignRT metrics - FBCT).

Dimension	Comparison pair	Median (mm)	95% CI	P
Lateral	FBCT vs. Real-time Mean	0.021	[-0.005, 0.048]	1.000 (NS)
	FBCT vs. Drift Mean	0.021	[-0.005, 0.049]	1.000 (NS)
	FBCT vs. Endpoint Drift	0.097	[0.050, 0.148]	0.001
Longitudinal	FBCT vs. Real-time Mean	-0.073	[-0.102, -0.044]	< 0.001
	FBCT vs. Drift Mean	-0.071	[-0.100, -0.042]	< 0.001
	FBCT vs. Endpoint Drift	-0.130	[-0.176, -0.082]	< 0.001
Vertical	FBCT vs. Real-time Mean	-0.235	[-0.273, -0.198]	< 0.001
	FBCT vs. Drift Mean	-0.238	[-0.275, -0.200]	< 0.001
	FBCT vs. Endpoint Drift	-0.351	[-0.415, -0.288]	< 0.001

**Figure 2:**
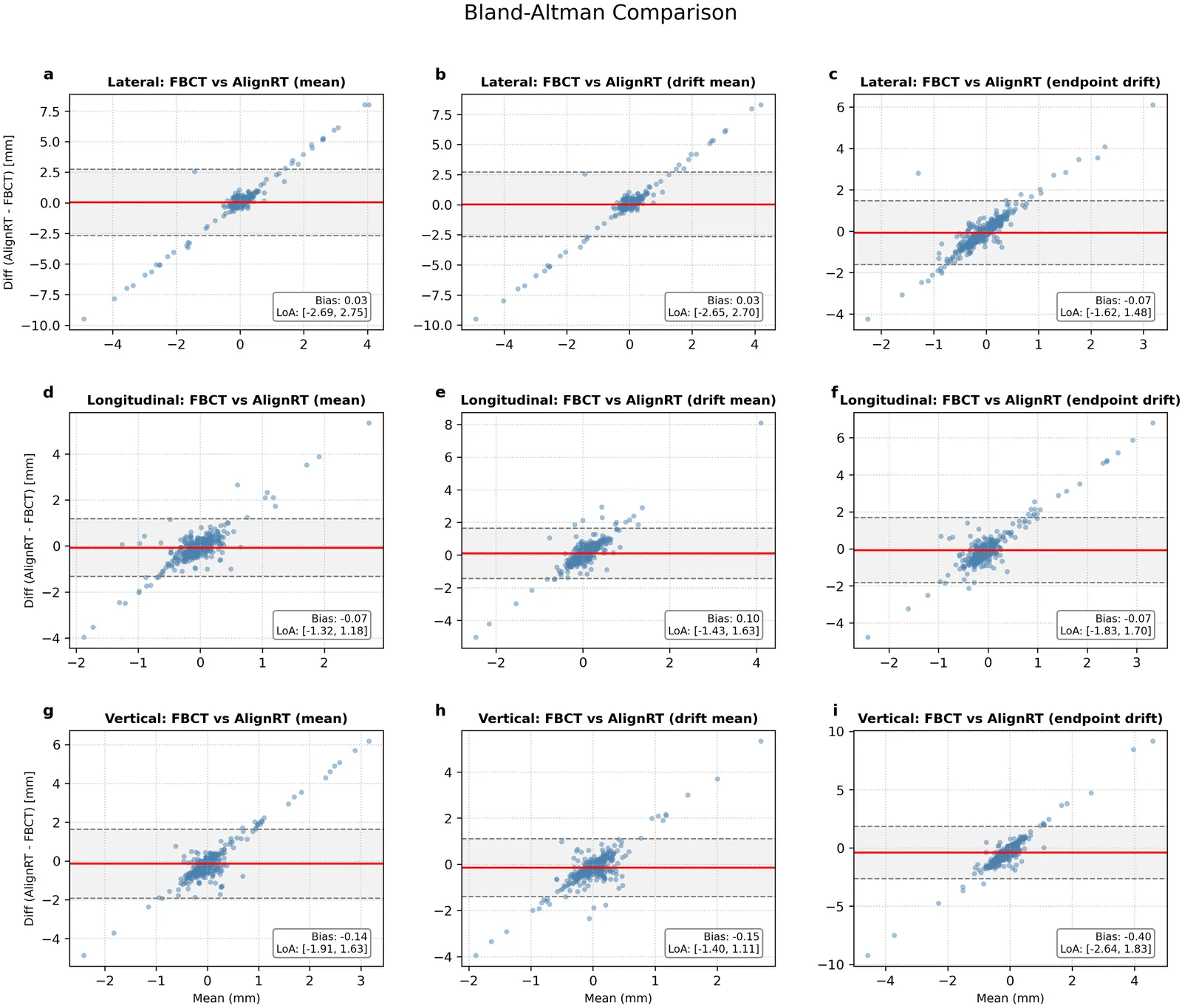
Bland-Altman comparisons between FBCT and AlignRT. **(a–c)** Lateral-axis comparisons using the real-time mean, mean baseline drift, and endpoint drift, respectively; **(d–f)** longitudinal-axis comparisons using the corresponding three AlignRT metrics; and **(g–i)** vertical-axis comparisons using the corresponding three AlignRT metrics. The solid red line represents the mean bias, and the dashed lines represent the 95% limits of agreement.

The clinical agreement between the two systems was subsequently assessed using Bland-Altman analysis (**Table 5**, **Figure 2**). The mean differences (Bias) across all metrics and dimensions were small, ranging from -0.40 mm to +0.10 mm, reflecting the absence of a substantial systematic offset. Lateral direction demonstrated the highest agreement. The 95% LoA for real-time mean and mean drift were approximately ± 2.7 mm. Notably, the endpoint drift exhibited the highest precision in this axis, with the LoA narrowing significantly to [-1.62, 1.48] mm. Longitudinal direction showed favorable agreement with LoA values, remaining within ± 1.8 mm across all metrics. Vertical direction exhibited the poorest agreement. While the bias for real-time mean was only -0.14 mm, it had a wider LoA ([-1.91, 1.63] mm). The endpoint drift showed the largest bias (-0.40 mm) and widest LoA ([-2.64, 1.83] mm), indicating that vertical monitoring is most sensitive to respiration-induced motion, leading to a lower stability in endpoint drift measurements compared with real-time means.

**Table 5:** Bland-Altman analysis summary.

Dimension	AlignRT metric	Bias (mm)	95% LoA (mm)
Lateral	Real-time Mean	0.03	[-2.69, 2.75]
	Drift Mean	0.03	[-2.65, 2.70]
	Endpoint Drift	-0.07	[-1.62, 1.48]
Longitudinal	Real-time Mean	-0.07	[-1.32, 1.18]
	Drift Mean	0.10	[-1.43, 1.63]
	Endpoint Drift	-0.07	[-1.83, 1.70]
Vertical	Real-time Mean	-0.14	[-1.91, 1.63]
	Drift Mean	-0.15	[-1.40, 1.11]
	Endpoint Drift	-0.40	[-2.64, 1.83]

### 3.4 Comparison of motion magnitudes across directions

To evaluate the relative stability of different motion planes, the RMS of intrafraction deviations was compared using the Friedman test, as presented in **Table 6**. Significant differences in motion magnitude were identified among the three translational axes (X^2^ = 227.369, P< 0.001). *Post-hoc* analysis with Bonferroni correction revealed that the LAT direction exhibited the smallest magnitude of instability (median RMS = 0.28 mm), which was significantly lower than both the LNG and VRT directions (P< 0.001). No significant difference was found between the LNG and VRT axes (P = 1.000), both of which showed approximately double the motion magnitude of the LAT axis. Similarly, the magnitude of rotational instability significantly varied across axes (X^2^ = 286.305, P< 0.001). Yaw rotation (median RMS = 0.21°) was identified as the most prominent rotational component, which was significantly larger than pitch (P< 0.001) and roll (P< 0.001). Furthermore, the magnitude of roll rotation was significantly smaller than that of pitch (P< 0.001), indicating that the patient setup remained most stable in the rolling plane.

**Table 6:** Comparison of intrafraction motion magnitudes across different dimensions.

Dimension		Median RMS	Interquartile range (IQR)	Friedman test P-value	Pairwise comparison (*Post-hoc*)
Translation	LAT	0.28mm	[0.17, 0.46]	**< 0.001**	LAT< LNG, VRT (P< 0.001)
	LNG	0.54mm	[0.38, 0.77]		LNG ≈ VRT (P = 1.000)
	VRT	0.52mm	[0.34, 0.86]		
Rotation	YAW	0.21°	[0.12, 0.35]	**< 0.001**	YAW > ROLL, PITCH (P< 0.001)
	ROLL	0.09°	[0.06, 0.16]		ROLL< PITCH (P< 0.001)
	PITCH	0.14°	[0.08, 0.26]		

Bold values indicate statistically significant overall or post-hoc comparisons (P < 0.05).

### 3.5 Factors influencing 6DoF motion stability

Linear mixed-effects models were employed to identify predictors of intra-fractional stability after 3SD outlier removal, in which major outputs are presented in **Table 7**. Respiratory frequency (F_res_) emerged as the most pervasive predictor for translational stability. Specifically, higher F_res_ was significantly associated with reduced magnitudes in both baseline drift (VRT: β = -0.061; LAT: β = -0.122; P< 0.001) and respiratory motion (VRT, LNG, and LAT: all P< 0.001), indicating that faster breathing patterns were correlated with diminished translational excursions. Conversely, monitoring duration was the primary driver of rotational instability. Prolonged duration significantly predicted a greater baseline drift across all rotational axes (roll, pitch, and yaw: β = 0.0007, P< 0.02) and a higher respiratory amplitude in the pitch dimension (β = 0.0001, P = 0.018), indicating a time-dependent degradation of rotational setup accuracy.

**Table 7:** Fixed-effect estimates (β coefficients) of clinical factors associated with 6DoF intra-fractional baseline drift or respiratory RMS.

Factors	VRT (mm)	LNG (mm)	LAT (mm)	ROLL (°)	PITCH (°)	YAW (°)
Baseline drift RMS						
F_res_ (Hz)	-0.061***	-0.003	-0.122***	-0.002	-0.0004	-0.004
Age (years)	0.009	0.001	0.014	-0.0003	0.002*	-0.001
Duration (s)	0.001	0.001	0.002	0.001*	0.001***	0.001*
Laterality (L vs R)	-0.317	-0.072	-0.287	-0.013	0.001	0.033
Fraction (No.)	0.024	0.001	0.031	-0.0002	-0.0003	0.002
Respiratory RMS						
F_res_ (Hz)	-0.031***	-0.005***	-0.076***	-0.001	-0.001	-0.002*
Age (years)	0.008	0.003	0.008	0.0000	0.0005	0.0001
Duration (s)	-0.0002	0.0001	-0.0000	-0.0001	0.0001*	-0.0001
Laterality (L vs R)	0.022	-0.002	0.016	0.007	0.008	0.017
Fraction (No.)	0.007	-0.001	0.010	0.0000	-0.0002	-0.0001

Statistical significance was indicated as *P < 0.05 and ***P < 0.001. Laterality reference level: Right side. All values represent fixed-effect estimates (β) derived from linear mixed-effects models, concentrating on the RMS of the baseline drift or intra-fractional respiratory signal after removal of 3SD outliers.

Regarding other clinical factors, older age was significantly associated with the elevated baseline drift in the pitch dimension (β = 0.0016, P = 0.044). Additionally, treatment fraction exhibited a consistently borderline trend (P = 0.050-0.068) toward increased translational drift and respiratory amplitude in later fractions for the VRT and LAT dimensions. Other factors, including patient laterality, did not reach statistical significance.

### 3.6 Non-linear temporal dynamics and stability breakpoints

**Figure 3:**
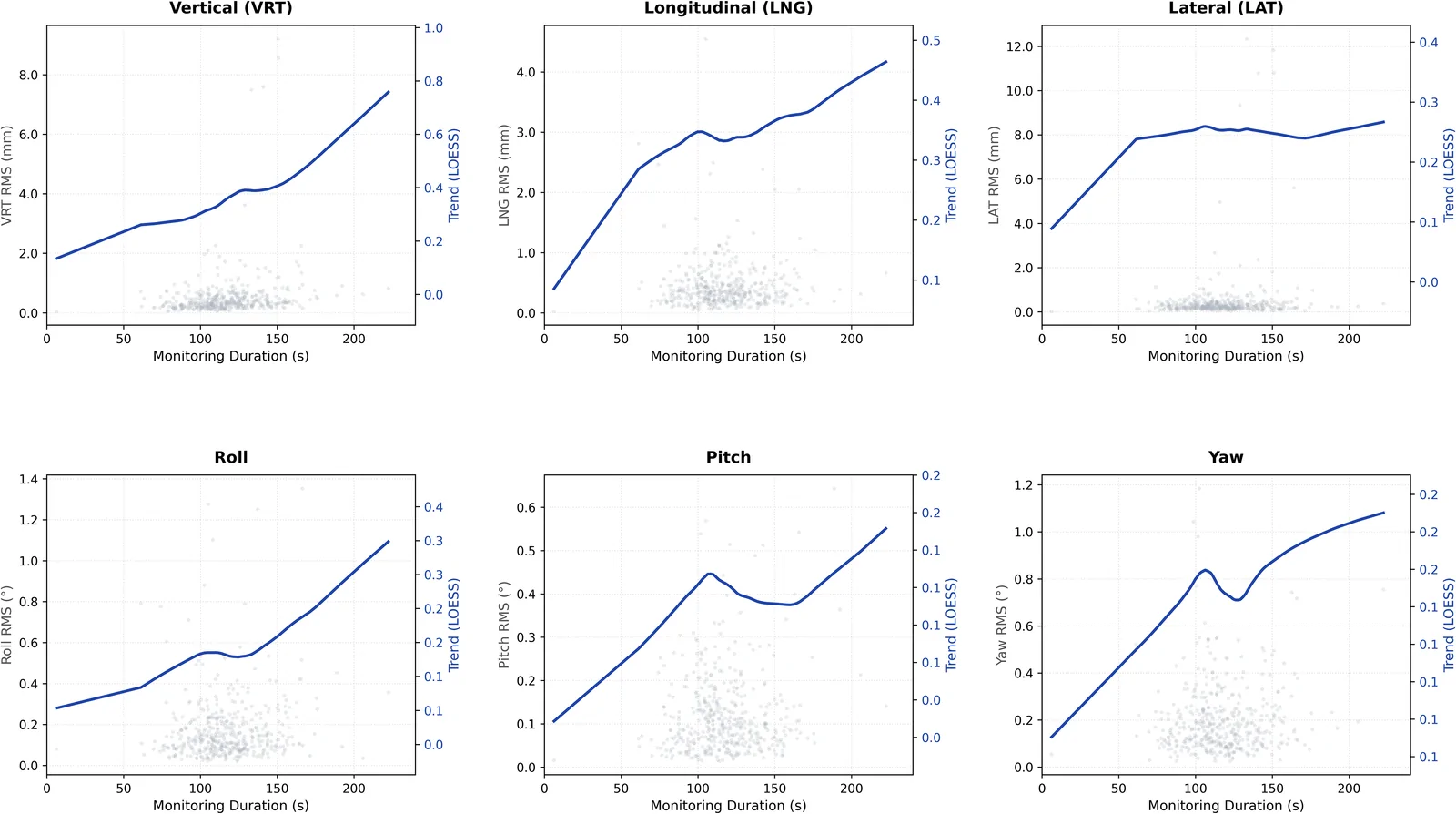
LOESS curves of baseline drift RMS across 6 dimensions. The sparse distribution of data points below ~50 s reflects the small number of fractions with a total monitoring duration shorter than 50 s (mean duration 121 ± 30 s); continuous surface monitoring was nonetheless performed from the start of every fraction, and the LOESS trend curves incorporate all data acquired throughout the monitoring period.

**Figure 4:**
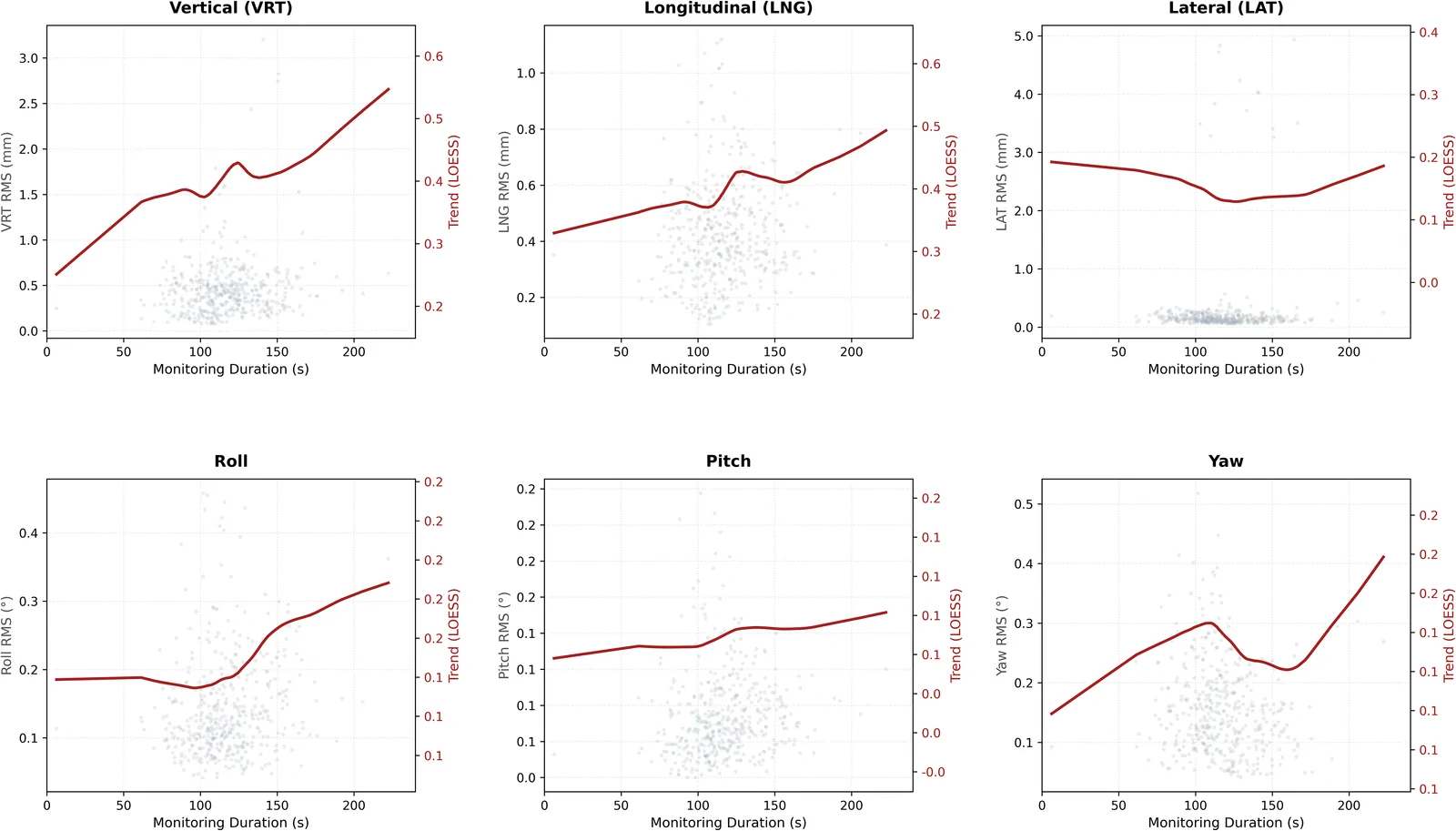
LOESS curves of respiratory motion RMS across 6 dimensions. The sparse distribution of data points below ~50 s reflects the small number of fractions with a total monitoring duration shorter than 50 s (mean duration 121 ± 30 s); continuous surface monitoring was nonetheless performed from the start of every fraction, and the LOESS trend curves incorporate all data acquired throughout the monitoring period.

LOESS regression and segmented linear modeling revealed significantly non-linear temporal dynamics in both baseline drift and respiratory motion, complementing the global findings of the linear mixed-effects models. As illustrated in **Figures 3**, **4**, a consistently “acclimatization phase” was found in LOESS curves during the initial 100 seconds of monitoring, where motion remained minimal or even slightly decreased. However, beyond this threshold, a distinct transition toward instability was identified across multiple dimensions, even in metrics, such as longitudinal drift, which did not reach statistical significance in global linear analysis.

For baseline drift (drift RMS, see **Table 8**), the segmented analysis identified optimal breakpoints (T_break_) clustered between 100.7s and 132.6s. During the initial stability period (T_break_), the drift rates (b_1_) were negligible, effectively remaining at zero. A subsequent accelerated degradation phase was characterized by an acceleration in drift magnitude, particularly in the lateral (LAT) dimension (b_2_ = 0.048). Notably, the pitch dimension demonstrated a statistically significant slope elevation after 132.6s (b_2_ = 0.002; 95% CI: 0.000–0.004), confirming a reliable time-dependent loss of rotational setup accuracy.

**Table 8:** Segmented regression (breakpoint) analysis of 6DoF baseline drift (Drift RMS).

Metric	Breakpoint Tbreak​ (s)	Initial slope b1​	Slope change b2​	95% CI of b2​	Status
VRT	102.8	0.001	0.030	[-0.013, 0.073]	Trend
LNG	116.8	0.001	0.012	[-0.001, 0.026]	Trend
LAT	100.7	-0.002	0.048	[-0.004, 0.101]	Trend
ROLL	115.0	0.000	0.005	[-0.002, 0.012]	Trend
PITCH	**132.6**	**0.001**	**0.002**	**[0.000, 0.004]**	**Significant**
YAW	123.2	0.000	0.004	[-0.001, 0.009]	Trend

Bold values indicate statistically significant slope changes, defined as a 95% CI of b2 that does not cross zero.

The respiratory motion (ResRMS, see **Table 9**) followed a strikingly synchronized temporal trajectory, and stability breakpoints were identified between 99.1s and 138.8s. Translational respiratory components remained highly stable during the first ~100 seconds before exhibiting increased excursions, most prominently in the VRT axis (b_2_ = 0.013). Similar to the drift findings, respiratory pitch reached a significant transition point at 138.8s (b_2_ = 0.002; 95% CI: 0.000–0.004). These patterns highlight that respiratory stability is a multi-phasic process, where a “stability window” is followed by a period of increased variability.

**Table 9:** Segmented regression (breakpoint) analysis of 6DoF respiratory motion (ResRMS).

Metric	Breakpoint Tbreak​ (s)	Initial slope b1​	Slope change b2​	95% CI of b2​	Status
VRT	101.4	0.003	0.013	[-0.016, 0.043]	Trend
LNG	111.4	0.002	0.006	[-0.003, 0.015]	Trend
LAT	99.1	0.001	0.004	[-0.002, 0.010]	Trend
ROLL	104.9	0.001	0.002	[-0.001, 0.005]	Trend
PITCH	**138.8**	**0.001**	**0.002**	**[0.000, 0.004]**	**Significant**
YAW	125.6	0.002	0.004	[-0.001, 0.009]	Trend

Bold values indicate statistically significant slope changes, defined as a 95% CI of b2 that does not cross zero.

## 4 Discussion

### 4.1 Comparison of FBCT and AlignRT for intrafractional validation

In this retrospective study of patients undergoing post-BCS, the agreement between AlignRT optical surface monitoring and FBCT measurements was systematically evaluated. Overall, the two systems did not exhibit substantial systematic offsets across translational dimensions, with mean biases ranging only from -0.40 mm to +0.10 mm. This indicates that continuous optical surface monitoring or surface guided radiation therapy (SGRT) possesses high reliability in tracking intrafractional motion, as highlighted by Hattel et al. (17) in their evaluation of setup and intrafraction motion for surface-guided whole-breast radiotherapy.

This study specifically introduced three AlignRT metrics (real-time mean, mean baseline drift, and endpoint drift) to comprehensively quantify motion characteristics. The results demonstrated significant performance-based differences among these metrics across dimensions, reflecting the physical reality of respiratory motion. In the LAT axis, which is least affected by breathing, the “real-time mean” and “mean drift” exhibited excellent agreement with FBCT, with a negligible median difference of 0.021 mm. This exceptional stability in the horizontal planes agreed with the findings from a large-scale, real-world analysis of 2028 breast cancer patients undergoing radiotherapy conducted by Reitz et al. (23) Notably, the internal correlation between AlignRT’s real-time mean and mean baseline drift was approximately perfect (rs​ > 0.99), confirming that both low-pass filtering (to extract baseline drift) and raw signal averaging effectively eliminated high-frequency respiratory oscillation noise. However, the “endpoint drift” exhibited significant deviations from FBCT in both LAT and VRT directions. This discrepancy arises because the endpoint drift constitutes only a single time-point positional measurement obtained at the conclusion of the monitoring period. Under free-breathing conditions, a limited-duration FBCT acquisition represents a time-averaged anatomical state over multiple respiratory phases, but it still cannot characterize the continuous temporal evolution of intrafractional motion. Transient respiratory variations and progressive baseline drift occurring before or after the scan cannot be captured. Piruzan et al. (7) highlighted the stringent requirements for effectively managing the complexities of periodic target motion in their review of breast cancer patients undergoing radiation therapy.

One of the most noticeable findings of this study was the highly significant (P< 0.001) while diametrically opposite systematic shifts recorded by FBCT and AlignRT in the VRT direction: FBCT indicated an anterior displacement (median 0.06 mm), whereas AlignRT captured a posterior drift (median -0.17 mm). This continuous “surface sagging” phenomenon directly supports the specific physiological observations made by Jensen et al. (4) in their investigation of intrafractional baseline drift during free-breathing breast cancer radiation therapy. This divergent motion trajectory directly accounts for the significantly negative median differences (range, -0.235 to -0.351 mm) found in the pairwise comparisons between AlignRT metrics and FBCT (**Table 4**). It was hypothesized that this “opposite shift” could be fundamentally driven by various anatomical targets tracked by the two systems. AlignRT monitors the external skin contour (soft tissue) of the patient’s chest. Over a monitoring duration averaging 121 s, breast soft tissue is prone to slight posterior sagging or relaxation due to gravity, muscle fatigue, or changes in skin tension. Conversely, FBCT alignment typically relies more heavily on internal bony landmarks (e.g., ribs and thoracic vertebrae). When the treatment beam turns off and the FBCT scan initiates, patients may subconsciously alter their breathing patterns, including taking a deeper breath due to relaxation or anticipation of the session ending, causing a subtle anterior expansion of the bony thorax. Furthermore, the VRT direction exhibited the widest LOA ([-2.64, 1.83] mm), confirming that the vertical axis is most significantly affected by thoracoabdominal respiratory motion. However, it should be emphasized that this internal–external decoupling remains a physiological *hypothesis* generated to explain the observed opposing vertical shifts, rather than a directly demonstrated mechanism. Because our measurements were confined to the external skin surface (AlignRT) and to single-timepoint internal bony verification (FBCT), we could not simultaneously observe the internal target and the external contour within the same temporal frame. Consequently, the proposed contribution of gravitational soft-tissue sagging relative to bony thoracic motion cannot be confirmed from the present data and should be interpreted with appropriate caution. Future studies incorporating simultaneous tracking with fluoroscopy or internal fiducial markers are warranted to elucidate the precise mechanisms of this internal-external anatomical decoupling (24–26).

Consequently, it is recommended that, when evaluating setup errors in horizontal planes (LAT and LNG) that are insensitive to respiration, endpoint drift remains a valuable metric and demonstrates the highest precision (e.g., LAT, [-1.62, 1.48] mm). However, when assessing the heavily respiration-influenced VRT axis, clinicians must strictly avoid relying on single-timepoint endpoint measurements. Prioritizing AlignRT’s real-time mean or the filtered mean drift effectively smooths pseudo-errors introduced by instantaneous respiratory excursions, thereby more accurately reflecting the patient’s actual intra-fractional baseline position. Previous research on three-dimensional surface scanning, conducted by Gaisberger et al. (21), indicated that such continuous monitoring is of paramount importance for ensuring accurate patient positioning during radiotherapy of breast cancer patients.

### 4.2 Temporal dynamics of intrafractional motion

To deeply investigate the complex mechanisms of intrafractional motion, this study innovatively applied a low-pass filtering technique to decouple raw 6DoF surface motion signals into baseline drift (representing slow postural shifts) and respiratory motion (representing rapid cyclic oscillations). This signal decomposition enabled us to independently evaluate the specific influences of various clinical factors on patient setup stability and breathing patterns. The results indicated a significant heterogeneity in postural instability across different axes. In translational dimensions, the LAT axis exhibited the highest stability (median RMS = 0.28 mm), whereas the LNG and VRT axes were approximately twice as large. This finding aligns with anatomical expectations, as diaphragmatic contraction primarily drives thoracic expansion in the superior–inferior (LNG) and anterior–posterior (VRT) directions, a directional susceptibility that has also been documented in large-scale analyses of intrafraction motion during breast cancer radiotherapy (23). Regarding rotational dimensions, yaw was identified as the most prominent axis of instability (median RMS = 0.21°). This is largely attributable to the specific setup requirements for post-BCS radiotherapy: patients typically present with the ipsilateral arm raised or hands clasped above the head. This asymmetric tension distribution predisposes the torso to compensatory left–right twisting (yaw) during treatment.

LMMs, applied after 3SD outlier removal, identified respiratory frequency (F_res_) as a critical clinical predictor and the most consistent predictor of translational stability. Specifically, a faster respiratory rhythm was significantly associated with a diminished baseline drift (VRT, LAT) and a reduced respiratory amplitude (VRT, LNG, and LAT). Biomechanically, for a given minute ventilation requirement, a higher respiratory frequency is typically correlated with a shallower tidal volume (shallow-fast breathing). As the spatial excursion of the diaphragm and chest wall is determined predominantly by tidal volume rather than by frequency, a reduced tidal volume physically constrains the expansion of the rib cage and the inferior displacement of the diaphragm within each respiratory cycle. This breathing pattern therefore physically constrains the expansion limits of the rib cage during a single respiratory cycle, macroscopically manifesting as smaller 3D spatial displacements. This finding indicates that, for FB patients who are unable to perform deep inspiration breath hold (DIBH), coaching them to maintain an even, slightly rapid, and shallow breathing pattern may help optimize target positional stability.

One of the most pivotal findings of this study is the highly non-linear temporal evolution of intrafractional motion. Globally, the LMM analysis confirmed that “monitoring duration” is the primary driver accumulating rotational errors, particularly increasing baseline drift in roll, pitch, and yaw. Subsequently, using LOESS smoothing and segmented (piecewise) nonlinear regression, the inflection points of this time-dependent degradation were precisely quantified. It was revealed that both baseline drift and respiratory motion exhibited an initial “acclimatization phase” lasting approximately 100 s, during which motion magnitudes remain minimal or may even slightly decrease. However, upon crossing the critical breakpoint (T_break_​, clustered between 99.1s and 138.8s), the magnitude of motion starts to accelerate adversely. For instance, baseline drift in the pitch dimension demonstrated a statistically significant slope increase after 132.6 s (P< 0.05), while translational drift (LAT) and respiratory excursions (VRT) also exhibited accelerated expansion trends following ~100 s. This multi-phasic pattern, characterized by an initial stability window followed by accelerated degradation, may reflect the progressive limitation of physiological postural compensation. Although gravitational loading can begin immediately after patient positioning, the observed intrafractional displacement represents the net result of passive tissue relaxation and active neuromuscular compensation. Patients undergoing breast radiotherapy maintain an extended arms-up posture, requiring continuous shoulder and trunk muscle activation. During the early treatment phase, this active compensation may partially counterbalance gravity-induced soft-tissue deformation, resulting in a relatively stable positional state. With prolonged immobilization, progressive fatigue and reduced postural control may diminish this compensation, potentially allowing relaxation-related deformation and baseline drift to accelerate. During the first 1.5–2 min of treatment, patients can rely on conscious effort and muscle tension to maintain the unnatural raised-arm posture. However, as time progresses, the accumulation of muscle fatigue, waning concentration, and potential positional discomfort (particularly in older patients, as the LMM analysis indicated that age was positively correlated with pitch drift), inevitably leads to involuntary postural relaxation and sagging. In the present cohort, the mean VMAT monitoring duration was 121 ± 30 s, placing it precisely at the trailing edge of the calculated “100–140 s stability window”. This indicates that highly efficient VMAT delivery duration in our cohort largely overlapped with this observed stability period, allowing dose delivery to complete before large-scale postural degradation occurs. However, if clinical protocols employ more time-consuming techniques, including static multi-field IMRT or complex non-coplanar plans, intrafractional errors may progressively increase. In such scenarios, integrating real-time surface monitoring or SGRT to continuously track patient motion, accompanied by proactive beam-hold interventions or timely repositioning, is strongly recommended to preserve dosimetric accuracy, as consistently supported by comprehensive surface-imaging studies (20).

This observation is also corroborated by Samadi Miandoab et al., who evaluated four external–internal correlation models (ECMs) and update strategies for estimating internal tumor motion from external respiratory surrogates (27). They demonstrated that, without model updates, the accuracy of internal-motion estimation progressively deteriorated as treatment time elapsed—primarily driven by baseline drift—whereas frequent baseline updating restored accuracy to near its initial level. This time-dependent decay is conceptually consistent with our finding of accelerating postural degradation after the ~100–140 s stability window and reinforces our recommendation for continuous, drift-adaptive monitoring during extended treatments. A key distinction, however, lies in treatment duration: their fractions lasted approximately 6–7 minutes, allowing baseline drift and irregular respiratory events to accumulate substantially, whereas our mean VMAT delivery was only 121 s—at the trailing edge of the stability window, before large-scale degradation typically manifests. This distinction reinforces our conclusion that the short delivery time of efficient VMAT largely completes dose delivery before the accelerated-motion phase, whereas time-dependent degradation becomes clinically critical predominantly for longer techniques (e.g., static multi-field IMRT or complex non-coplanar plans). Notably, Samadi Miandoab et al. also found that higher-order models were less robust to irregular respiration—including transient events such as deep breaths or coughing. In our cohort, we did observe such non-steady-state respiratory events, which were identified as transient motion artifacts and excluded prior to modelling; moreover, the relaxation-associated deeper breathing we hypothesized to underlie the divergent vertical (anterior) shift near the end of the fraction represents precisely this type of pattern. For patients exhibiting such relaxation-induced deep breathing or other irregular patterns, we therefore recommend reinforced breathing coaching together with continuous surface monitoring and drift-adaptive re-verification or beam-hold triggers, rather than reliance on a static pre-treatment gating model, so as to preserve gating accuracy.

More broadly, recent advances in real-time motion management have increasingly focused on improving the estimation of internal tumor motion through sophisticated correlation models and multimodal imaging. Representative developments include fluoroscopic image-based tumor tracking, adaptive external–internal correlation models, deep learning-based correlation frameworks (e.g., CNN-GRU architectures), and MRI-guided real-time target tracking (28–30). While these approaches differ substantially in imaging modality and computational methodology, they share a common objective: maintaining accurate internal target localization despite respiratory variability and baseline drift. In contrast, the present study addresses a complementary aspect of motion management by characterizing the temporal evolution of the external surface signal itself. Our findings regarding the physiological stability window, baseline drift, and respiratory dynamics provide quantitative evidence that may facilitate the development of more robust adaptive correlation models and improve the reliability of surface-guided motion management, particularly during prolonged treatments.

Beyond the global amplitude and RMS metrics employed here, respiratory motion in free-breathing patients can exhibit substantial irregularity, including intra-fraction variations in breathing position and amplitude, baseline shifts, and changes in waveform shape and frequency. Such deviations from idealized periodic breathing are common in clinical practice and are clinically important because they may substantially compromise treatment accuracy: amplitude and baseline variations enlarge the effective motion envelope, whereas waveform and frequency changes can reduce the robustness of respiratory correlation models and motion management strategies, as demonstrated by Samadi Miandoab et al. (27). In our cohort, transient non-steady-state events, such as coughing, swallowing, deep breaths, or sudden postural adjustments, were occasionally observed and were identified as transient motion artifacts and excluded from quantitative modelling. In addition, the relaxation-associated deeper breathing hypothesized to underlie the divergent vertical shift near the end of treatment may represent one manifestation of this type of irregular respiratory behavior.

From a clinical management perspective, these findings provide a quantitative basis for incorporating time-dependent motion characteristics into intrafractional management. Current breast radiotherapy workflows commonly rely on pre-treatment image guidance and predefined setup margins; however, these approaches provide limited information regarding posture evolution during beam delivery. Continuous SGRT monitoring enables clinicians to assess whether patients remain within an acceptable stability range throughout treatment rather than evaluating positional accuracy only at isolated time points. Several practical strategies can be supported. First, structured breathing coaching before and during treatment may promote a more regular and shallow breathing pattern, thereby reducing amplitude and baseline variability. Second, continuous real-time surface monitoring of baseline drift, rather than reliance on a static pre-treatment reference, facilitates the early detection of progressive or irregular deviations. Third, when persistent baseline drift or repeated tolerance excursions are observed, re-baselining or patient repositioning should be considered. For patients with unstable breathing, tighter gating thresholds and, when necessary, temporary beam interruption may further help preserve gating performance and geometric accuracy. A comprehensive time-resolved and frequency-domain characterization of irregular respiratory patterns—and their differential effects on real-time mean displacement, mean drift, and endpoint drift throughout treatment—remains an important direction for future investigation.

Recent work by Galmiche et al. (31) also provides a complementary perspective on breast surface changes during radiotherapy. Using a functional-map framework applied to 3D surface scans, they characterized breast shape and volume changes throughout postoperative breast radiotherapy, reporting mean inter-fractional surface displacements of approximately 1–2 mm and relative volume changes of up to 10% (31). Importantly, the two studies address different temporal scales and are therefore complementary rather than directly comparable. Galmiche et al. focused on gradual inter-fractional anatomical deformation driven by biological processes such as seroma involution and edema, whereas our study used continuous AlignRT monitoring to characterize intra-fractional respiratory motion and postural drift during beam delivery. Nevertheless, both studies indicate that the magnitude and clinical relevance of surface changes increase with the observation timescale. Together, these findings support an integrated surface-guided monitoring strategy, in which continuous intra-fractional tracking maintains geometric accuracy during treatment, while periodic inter-fractional surface assessment facilitates the detection of cumulative anatomical changes that may ultimately support adaptive radiotherapy. Recent technological advances further suggest that surface-guided imaging will increasingly be integrated with AI-assisted motion prediction and adaptive radiotherapy platforms, potentially enabling more personalized respiratory motion management (32).

Overall, building on the identified ~100–140 s stability window, we propose a tiered monitoring-and-intervention framework, presented as a data-informed starting point rather than a universal standard that centers may adapt to their own margins and dosimetric tolerances. Within the initial stability period (first ~100–140 s), continuous monitoring should apply a 2 mm/2° warning threshold, prompting attention and breathing coaching; once drift exceeds 3 mm or 3° in any axis, the beam should be held for repositioning. As the vertical (VRT) axis is most respiration-sensitive, intervention here should rely on the filtered mean drift or real-time mean rather than a single instantaneous value. For deliveries extending beyond ~140 s—especially long techniques such as static IMRT or complex non-coplanar plans—we recommend tightening the threshold to 2 mm/2°​ with a mid-fraction verification, given the accelerated degradation after the breakpoint. Being derived from a single-center cohort, these thresholds warrant prospective validation.

A major challenge in quantifying intrafractional motion from SGRT data is accurately stripping away high-frequency respiratory oscillations to extract the true baseline drift. Previous similar studies (e.g., Delombaerde et al. (22)) have frequently employed a “moving average” or “windowed average” method with extensive intervals [e.g., 60 s (22)] to smooth respiratory curves. The moving-average approach suffers from inherent limitations. Although a large temporal window can average out breathing cycles, it severely compromises the temporal fidelity of the signal because of pronounced phase delays, causing the extracted baseline drift to lag and become blurred along the time axis. In contrast, this study applied a second-order zero-phase Butterworth low-pass filter. The cutoff frequency (f_c_) was set at 0.05 Hz, corresponding to a 20-s period. This threshold was selected to effectively eliminate typical adult respiratory frequencies (approximately 12–20 breaths/min, or 0.2–0.3 Hz), thereby enabling a clean separation of slow postural shifts D (t) from cyclic respiratory oscillations R (t). Importantly, the zero-phase property ensures that the filtered signal experiences no temporal shift or lag. Preserving temporal fidelity is critical for the dynamic analyses performed in this study. Because the filtering introduced no phase distortion, the segmented regression models were able to detect subtle nonlinear trajectory changes and accurately identify the timing of stability breakdowns (T_break_), which clustered between 100.7 s and 138.8 s. If a conventional moving-average filter had been used, these slope inflections might be obscured by coarse smoothing, and phase delays could lead to an overestimation of the duration of the “stability window.” Moreover, this high-quality signal separation enabled accurate calculation of respiration RMS, which allowed us to independently verify the biomechanical constraining effect of respiratory frequency (F_res_) on spatial displacement in the LMM analysis.

Several limitations of this study should be acknowledged. Firstly, as a single-center retrospective analysis, the sample size was relatively small (38 patients, involving 485 validated fractions) and included only female post-BCS patients treated with VMAT under FB conditions. Although the unavailable AlignRT recordings were caused by workflow-related technical issues rather than patient-specific factors, these missing data were more frequent during the early phase of data collection. Therefore, a potential selection bias due to incomplete data acquisition cannot be completely excluded. Additionally, although mixed-effects models were applied to account for within-patient correlation, the number of available fractions varied among patients, and unequal contribution of individual patients may introduce potential weighting effects in fraction-level analyses. Secondly, the generalizability of the nonlinear temporal dynamics, such as the 100–140 s “stability window”, to other radiotherapy techniques (e.g., DIBH or longer static IMRT deliveries) or to other anatomical sites requires further validation through large-scale, multicenter prospective studies. Because most VMAT fractions in our cohort were completed within approximately 2 minutes, dedicated long-duration monitoring datasets are required to determine whether the identified transition point remains consistent during extended treatment sessions. Although segmented regression was performed using continuous time-resolved measurements rather than duration-stratified groups, the possibility of reduced sampling density influencing breakpoint estimation cannot be fully excluded. The temporal availability of fraction-level observations and patient representation across monitoring duration is shown in **Supplementary Figure 3**. Future studies involving prolonged treatment sessions (e.g., 5–10 min monitoring periods) are required to validate whether the observed 100–140 s transition represents a general physiological phenomenon. Thirdly, and most importantly, this study lacked concurrent continuous internal imaging to synchronously track internal anatomy during treatment; the anatomical-decoupling explanation for the divergent vertical shifts between AlignRT and FBCT therefore remains an unverified hypothesis. Direct confirmation will require study designs that capture internal and external motion simultaneously and continuously. In addition, the present analysis primarily characterized global respiratory motion using peak-to-peak amplitude and root-mean-square (RMS) metrics. More detailed characterization of complex respiratory dynamics—including breath-to-breath variability, temporal evolution of respiratory patterns, frequency-domain characteristics, and baseline-drift behavior throughout treatment—was beyond the scope of the present study. Although transient irregular respiratory events, such as deep breathing or coughing, were occasionally observed during treatment, they were treated as motion outliers rather than systematically quantified. Given the relatively large cohort of 485 validated fractions, rigorous investigation of these time-resolved respiratory characteristics will require dedicated methodological development and comprehensive validation, which we plan to pursue in future studies. Specifically, future validation studies could employ: (i) real-time kV/MV fluoroscopic tracking of implanted internal fiducial markers concurrent with AlignRT surface monitoring, enabling direct temporal correlation between external soft-tissue displacement and internal skeletal/target position; (ii) MRI-linac platforms providing continuous, radiation-free volumetric visualization of internal anatomy alongside surface signals; and (iii) 4D-CT or repeated intrafractional CBCT synchronized with surface traces to quantify the phase and amplitude relationship between the external contour and internal bony landmarks. Such multimodal, temporally co-registered datasets would allow the decoupling hypothesis to be tested quantitatively and, if confirmed, would inform surface-to-internal motion models that improve the dosimetric interpretation of SGRT signals.

## 5 Conclusions

This study demonstrated that AlignRT monitoring exhibited high clinical reliability in FB radiotherapy following BCS, demonstrating good agreement with FBCT in the horizontal dimensions. More importantly, compared with the single-timepoint snapshot provided by FBCT, AlignRT continuously tracks and captures dynamic baseline drift and potential postural sagging over time, particularly in the vertical dimension, thereby providing a valuable and indispensable complement to conventional radiographic verification. Furthermore, this study revealed remarkable nonlinear temporal dynamics in intra-fractional motion. A faster, shallow breathing pattern may help constrain 3D spatial displacement. In addition, a stability window of approximately 100–140 s was identified, beyond which motion degradation accelerates. Although the delivery time of conventional VMAT typically falls in this initial stability period, the routine integration of continuous optical surface monitoring and timely intervention remains essential to maintain dosimetric accuracy, particularly for complex techniques that require longer treatment durations.

## Data Availability

The original contributions presented in the study are included in the article/**Supplementary Material**. Further inquiries can be directed to the corresponding author.

## Supplementary material

Supplementary Figure 1 Distribution of the mean intrafractional respiratory frequency across all 485 analyzed fractions. The observed range (8.6–26.2 breaths/min; 0.143–0.437 Hz) lies entirely above the 0.05 Hz low-pass cutoff used for baseline–respiratory signal decomposition, with the minimum value exceeding the cutoff by a factor of approximately 2.9.

Supplementary Figure 2 Flow diagram of fraction selection. Of 687 initially screened treatment fractions from 41 patients, 202 were excluded during data-quality screening (missing AlignRT data, n = 104; incomplete scan logs, n = 89; mismatched timestamps between the FBCT and AlignRT systems, n = 9), yielding 485 validated fractions from 38 patients (17 left-sided, 264 fractions; 21 right-sided, 221 fractions) for final analysis.

Supplementary Figure 3 Temporal availability of fraction-level observations and patient representation during AlignRT monitoring. **(A)** Number and percentage of fractions with available monitoring data at each elapsed time point. A fraction was considered available at time if its monitoring duration was ≥ *t*. **(B)** Number and percentage of unique patients represented at each elapsed time point. A patient was considered represented at time *t* if at least one of her treatment fractions had a monitoring duration ≥ *t*; thus, patient-level availability was determined by the maximum monitoring duration across all fractions rather than by the mean monitoring duration. The shaded area indicates the identified stability window (100–140 s). Values at 100, 120, and 140 s are annotated.
